# Assessing lipid‐lowering and plasma cholesteryl ester transfer protein activity of *Centranthus longiflorus* and β‐Sitosterol following administration to triton WR1339‐ treated rats

**DOI:** 10.1002/fsn3.4471

**Published:** 2024-10-24

**Authors:** Seda Askin, Fatma Zuhal Umudum

**Affiliations:** ^1^ Health Services Vocational School Ataturk University Erzurum Turkey; ^2^ Medicine Faculty Ataturk University Erzurum Turkey

**Keywords:** *Centranthus longiflorus*, cholesteryl Ester transfer activity, hyperlipidemia, triton WR‐1339, β‐Sitosterol

## Abstract

The aim of this study was to evaluate the lipid‐lowering and plasma cholesteryl ester transfer protein(CETP) activity of *Centranthus longiflorus*(CL) and β‐Sitosterol(βS) following intraperitoneal administration of Triton‐WR 1339 (=Tyloxapol) (TWR) to male Wistar rats. Hyperlipidemia(HL) was developed by intraperitoneal injection of TWR. The animals were divided into main eight groups of six rats each. Rats were housed in separate cages and fed a standard diet for 7 days. After 7 days, ethanol extraction of CL plant, aqueous suspension of βS and anacetrapib was given to rats by oral gavage 1 h before the triton injection. Blood samples were collected and used for the biochemical parameters analysis. Histopathological studies were also performed on liver tissue. In hyperlipidemic rats(HR), CL extract and βS reduced total cholesterol similarly. βS lowered low‐density lipoprotein(LDL‐C) more than CL extract. Both CL extract and βS approximated impaired Alanine transaminase(ALT) and Aspartate transaminase(AST) levels in HR's to the level of control. CL extract provided better protection than βS against deterioration in liver tissue samples seen in hyperlipidemic rats. Finally, CL extract and βS inhibited CETP, at which point βS was more effective. These findings showed that CL extract and βS reduce plasma lipid concentration and may have a hypolipidemic effect due to their anti‐CETP properties.

## Graphical abstract

This study was aimed to evaluate in vivo the lipid‐lowering and plasma cholesteryl ester transfer protein activity of *Centranthus longiflorus* and β‐Sitosterol. *C. longiflorus* species has medicinal plant properties due to some natural metabolites (such as β‐ sitosterol) it contains. CETP inhibitors increase HDL‐C as well as lower LDL‐C. CL and βS caused inhibition of CETP both when administered alone and in combination with triton WR‐1339.

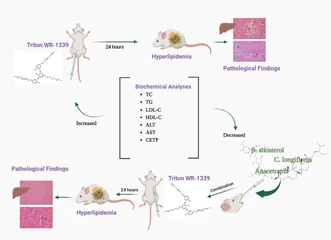

## 1 INTRODUCTION

Recently, a wide range of comorbid conditions have been associated with hyperlipidemia (HL), including cardiovascular diseases (coronary artery disease, atherosclerosis, heart failure, myocardial inflammation, etc.), obesity, type 2 diabetes, and non‐alcoholic fatty liver disease. Statins, which inhibit 3‐hydroxy‐3‐methylglutaryl coenzyme A reductase (HMG‐CoA reductase), which acts as the main enzyme in cholesterol synthesis from past to present, is used as anti‐hyperlipidemic drugs to reduce heart problems and death (Douglas & Channon, 2014). Statins reduce cardiovascular mortality and morbidity as well as cardiovascular events by lowering low‐density lipoprotein cholesterol (LDL‐C) levels, especially in patients at very high risk for cardiovascular disease. Although they are considered very safe drugs, there are many studies that, due to their wide use, their side effects may overshadow their proven beneficial effects (Simic & Reiner, 2015). The most important side effects of statins are neuromuscular diseases such as myopathy and rhabdomyolysis (Attardo et al., 2022). In addition, rare and usually reversible liver enzyme abnormalities have also been reported (Gillett Jr & Norrell, 2011). Another side effect is that they slightly increase the incidence of type 2 diabetes mellitus (Galicia‐Garcia et al., 2020). Statin therapy has also been associated with renal disease progression (Mansi et al., 2022). While there are concerns that statins may increase cancer, cognitive dysfunction, and memory loss, there is no satisfactory evidence that they cause such diseases (Rojas‐Fernandez et al., 2015).

In the last few decades, a number of new molecules and strategies have been developed for the control of cholesterol. For all these side effects of statins, the limited potential of other alternatives such as fibrates, bile acid sequestrants and niacin has prompted the search for new drug molecules with better efficacy and safety. CETP is a liver‐synthesized glycoprotein whose main functions are facilitating the transfer of both cholesteryl esters from HDL‐C particles to apolipoprotein B (apoB)‐ containing particles as well as the transfer of triglycerides from apoB‐containing particles to HDL particles (Nurmohamed et al., 2022). CETP inhibitors such as evacetrapib and anacetrapib are recognized as new agents that increase HDL‐C as well as lower LDL‐C (George et al., 2015). Studies are ongoing to reveal the advantages of these new molecules and strategies over conventional therapy in lipid management (Schmidt et al., 2021). Initially developed CETP inhibitors (torcetrapib, dalcetrapib) were not found to be effective enough in the management of high cholesterol. Next‐generation CETP inhibitors (anacetrapib, evacetrapib) were reported to be more effective and safe, especially in reducing LDL‐C and apoB and increasing HDL‐C (Nurmohamed et al., 2022). Anacetrapib has been shown to reduce the risk of atherosclerotic cardiovascular disease and also reduce the risk of new‐onset type 2 diabetes, improving glucose tolerance and insulin sensitivity (Bowman et al. 2017; Masson et al., 2018). In conclusion, the positive findings summarized above have increased the interest in CETP inhibitors. Today, studies have focused on herbal products and their natural compounds that may have the potential to be CETP inhibitors in the management of high cholesterol. This strategy is a useful model developed against the existing side effects of statins (Mohammadpour & Akhlaghi, 2013).

*Centranthus* genus is herbeous and shrub‐shaped plant belonging to the Caprifoliaceae family and consists of about 11 species, distributed mainly in the Mediterranean and Europe (Das et al., 2021; Tsymbalyuk et al., 2021). *C. longiflorus* (CL) species have medicinal plant properties due to some natural metabolites it contains (Suleyman et al., 2007). Cyclopentanes, monoterpenes, valepotriates, antioxidant pigments, two kinds of steroids, flavonol glycosides and glyceric acids were found in their phytochemical content (Kuruüzüm‐Uz et al., 2002).

In a previous study, the average amount of βS in 10 mg/mL plant powder of CL was determined as 226.603 ± 9.412 μg and the recovery rate was 2.26% (Askin et al., 2018). This value was the most concentrated compound we detected in this plant content. Therefore, we designed this study on both the plant extract and beta‐sitosterol, which is the most abundant of this plant ingredient.

Phytosterols are a subclass of steroids, a basic class of bioorganic molecules. Phytosterols found in a wide variety of organisms, including plants, animals, and fungi, exhibit structural similarity to cholesterol. These natural metabolites are the primary component of cholesterol found in animal cell membranes. They also affect cell membrane elasticity and serve as a secondary messenger in developmental signaling. Because of these properties, they are indispensable for organisms. A number of studies have found that those who eat a diet rich in phytosterols have a significantly reduced risk of cancer (for example, in Asian populations compared to Westerners) (Wang et al., 2021, 2023). βS is the most abundant phytosterol and in nearly all plant foods. It has been used in the treatment of many diseases due to its anti‐inflammatory, antipyretic, analgesic and antidiabetic effects (Wang et al., 2019). Recently, there are studies on the cholesterol‐lowering effect of βS (Chen et al., 2020; Wang et al., 2019).

Today, the main research topics that attract the attention of scientists and are focused on more are the plants that are found naturally in nature and whose effects have not yet been fully discovered, and the active substances that these plants contain. It is very important to reveal the action mechanisms of these plants and the bioactive compounds they contain, which can be good for diseases. In addition, the question of whether the plant extracts themselves or the bioactive compounds they contain are effective in curing these diseases is also on the agenda. This is one of the questions we aim to answer in this study.

Regardless of the wide variety of phytochemicals, only a few reports on pharmacological investigations of this species are available (Suleyman et al., 2007). Therefore, our research was designed to evaluate the safety profile and lipid‐lowering potential of the ethanolic extract from the aerial parts of CL and the abundant βS in this plant ingredient. Thus, with the results obtained, it was revealed that a plant itself or the active ingredient it contains is more effective in managing cholesterol.

## 2 MATERIALS AND METHODS

### 2.1 Plant material

The CL plant was collected from the Tortum district of Erzurum province, located in the northeastern Turkey, at the end of spring. Botanical identification was carried out by Prof. Dr. Mehmet Nuri Aydogan (Department of Biology, Faculty of Science, University of Ataturk) according to the Richardson (Richardson, 1975). The above‐ground parts of the plant were shade‐dried, powdered and stored in a tightly closed container for further use, at room temperature.

### 2.2 Preparation of ethanolic extract from the aerial parts of CL

In the study, different solvents such as ethanol, methanol, ethyl acetate and chloroform were used for the extraction of plant material. Ethanol was preferred as the solvent because non‐significant differences were observed when the plant contents were analyzed. In this method, 100 g of dry CL was taken and 200 mL of ethanol was added to it in a glass jar and left to shake for 72 h at room temperature. At the end of the period, the sample was filtered using filter paper. Ethanol was removed from the filtrate using a rotary evaporator. After removing the ethanol, all the dry extract was scraped off (Kotan et al., 2010). 21 g of extract was obtained as a result of ethanol extraction from 100 g of dried CL. The ethanolic extract was the most polar extract, with low toxicity for that reason it was used in the in vivo study. Application solution was prepared at doses of 150 mg/kg for administration to rats, and 1 mL of gavage was given to each animal in the treatment groups (Zengin et al., 2016).

### 2.3 β‐ Sitosterol (βS)

The active ingredient of βS was purchased from Santa Cruz Biotechnology (USA). In the relevant treatment groups, 0.4% βS was added to the diet of rats (Feng et al., 2018). In the study, 0.5% (w/v) CMC (Carbonymethylcelulose) was used to prepare the aqueous suspension of βS. The sample was sonicated for 30 min at 40°C.

### 2.4 Anacetrapib (ACP)

ACP was purchased from Merck. ACP was given orally to rats fasted for 12 h at 0.2 mg/mL, 2.5 mL/kg, o.d (Tan et al., 2010).

### 2.5 Animals, induction of hyperlipidemia and biochemical parameters

This study was carried out with the approval decision of Atatürk University Medical Experimental Application and Research Center ethics committee numbered 42,190,979‐000‐E.1700224647 and were in accordance with the internationally accepted principles for laboratory animal use and care. In this study, 48 male Wistar rats (average weight: 250–350 g, average age: 10–12 weeks old) taken from Atatürk University Experimental Animal Unit were used. The rats were kept in groups of six in polycarbonate cages and on a 12 h light/dark cycle at room temperature (22 ± 2°C) with free access to food and water until the start of the experiment. Throughout the experiment, the animals were given enough (ad libitum) water and pellet food (composition of standard diet for rats: moisture 6.00%, crude protein 18.40%, crude fat 4.35%, crude fiber 3.15%, calcium 1.10%, phosphorus 0.50%, total ash 4.50%, carbohydrates 62.00%). Animal experiments and procedures the use and care of laboratory animals were carried out in accordance with the rules specified of the international guidelines (Cinar et al. 2019). All applications were made to animals fasted for 12 h. 24 h after the applications, the animals were anesthetized according to the order of administration. Intracardiac blood samples and liver tissues were taken.

HL was experimentally induced by intraperitoneal injection of TWR (2.5 mL/kg) in rats fasted for 12 h. After approximately 24 h, the animals were considered hyperlipidemic. TWR was purchased from Santa Cruz Biotechnology (USA). Male Wistar rats that are 10–12 weeks old and weighing 250–350 g were divided into eight groups consisting of six rats each and treated as follows:

Group 1: Healthy control (HC) group, received intraperitoneal administration of normal saline and water by gavage.

Group 2: Anacetrapib (Reference) (ACP) group, received ACP (0.2 mg/mL, 2.5 mL/kg, o.d) 30 min before physiological water (2.5 mL/kg, i.p) administration.

Group 3: Triton WR‐1339 (Hyperlipidemia) (TWR) group, received physiological water 30 min before TWR (400 mg/kg, 2.5 mL/kg, i.p) administration.

Group 4: *C. longiflorus* (CL) group, received plant extract [150 mg/kg, oral dose (o.d)] 30 min before physiological water (2.5 mL/kg, i.p) administration.

Group 5: β‐ Sitosterol (βS) group, received βS (0.4%, o.d) 30 min before physiological water (2.5 mL/kg, i.p) administration.

Group 6: *C. longiflorus* + Triton WR‐1339 (CL + TWR) group, received plant extract (150 mg/kg, o.d) 30 min before TWR (400 mg/kg, 2.5 mL/kg, i.p) administration.

Group 7: β‐ Sitosterol + Triton WR‐1339 (βS + TWR) group, received βS (0.4%, o.d) 30 min before TWR (400 mg/kg, 2.5 mL/kg, i.p) administration.

Group 8: Anacetrapib + Triton WR‐1339 (ACP + TWR) group, received ACP (0.2 mg/mL, 2.5 mL/kg, o.d) 30 min before TWR (400 mg/kg, 2.5 mL/kg, i.p) administration.

Rats were anesthetized with sevoflurane. The blood samples were collected after 24 h of the applications in all experimental groups and placed on ice. Plasma samples were obtained by centrifugation at 4000 rpm and + 4°C for 10 min. The obtained plasma samples were stored at −80°C until analysis. Total cholesterol (TC), Triglyceride (TG), LDL‐C, HDL‐C, ALT, AST and CETP values were measured. Enzymatic color test for the quantitative determination of TC, TG, LDL‐C and HDL‐C in the plasma of animals on Beckman Coulter AU analyzers, kinetic UV test for the quantitative determination of ALT and AST, and CETP Activity for the quantitative determination of CETP Assay Kit (Sigma, Catalog No: RBG3148V) was used.

The liver tissues were rinsed with cold normal saline to remove blood contaminant and then immediately immersed in liquid nitrogen and stored at −80°C until further histopathological assessment. Finally, tissue samples were fixed directly in a 10% buffered formalin phosphate solution for histopathological evaluation.

### 2.6 Histopathological examinations

A piece of tissue from the liver was preserved in formalin solution (10%) for 24 h. Then, the samples were washed using tap water and dilutions of methyl, ethyl and absolute ethyl were applied for dehydration. Specimens were purified in xylene and embedded in paraffin at 56°C in a hot air oven for 24 h. Paraffin beeswax tissue blocks were prepared for portioning with a thickness of 4 μm by a sledge microtome. The acquired tissue portions were accumulated on glass slides, deparaffinized, and stained by hematoxylin and eosin stain for routine examination. Then, the examination was conducted with a light microscope Olympus BX53 model UIS 2 optical system. At least five microscopic areas were evaluated to score the specimen. The criteria for liver injury were hydropic degeneration, vascular congestion and sinusoidal dilatation. Each specimen was scored using a scale (−: none, +: mild, ++: moderate, and +++: severe) for injury, semiquantitatively (Askin et al., 2022).

### 2.7 Statistical analyzes

IBM SPSS Statistics for Windows, version 20.0 (IBM Corp.) package program was used for statistical evaluation of the data. First, descriptive statistics were calculated. The normality of the data was analyzed by the Kolmogorov–Smirnov test and the relationship between groups was analyzed by One way Anova. Analysis of Variance was applied according to the data obtained from the groups. After the analysis, the Tukey test, one of the Multiple Comparison Tests, was applied in case the variances were homogeneously distributed, and the Tamhane test was applied in case of not homogeneous distribution. Data were expressed as mean ± standard deviation. The *p* < .05 value was accepted for the lowest statistical significance level between the group means.

## 3 RESULTS

### 3.1 Serum lipid profiles

**FIGURE 1:**
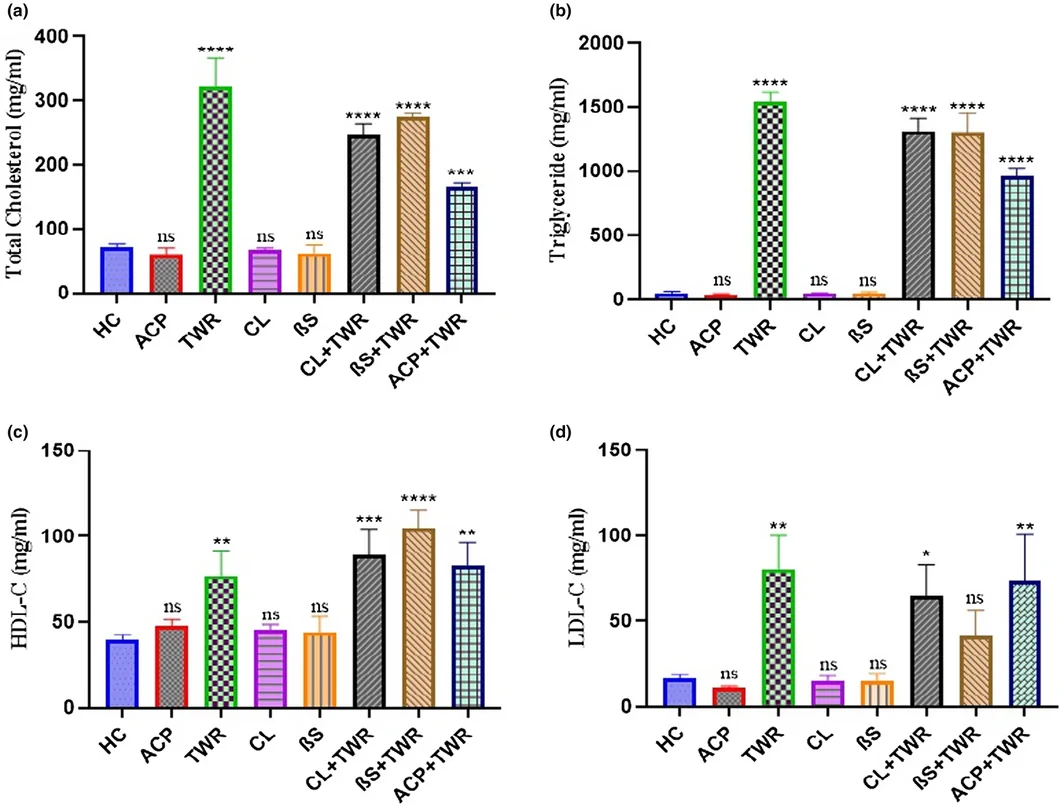
Effects of Triton Wr‐1339 (TWR), anacetrapib (ACP), *C. longiflorus* (CL) and β‐sitosterol (βS) on plasma lipid levels in Wistar rats. (a) total cholesterol (TC), (b) triglyceride (TG), (c) high‐density lipoprotein (HDL)‐ cholesterol, (d) low‐density lipoprotein (LDL)‐ cholesterol levels from plasma of Wistar rats (Tukey's multiple range tests were used); *significant changes between control and treatment groups (ns: Not significant, **p < .01, ***p < .001, ****p < .0001).

In the study of total lipid content, TC, TG, HDL‐C and LDL‐C levels belonging to eight different groups were determined. The results of these groups are given in Table 1 and Figure 1.

**TABLE 1:** Levels of total cholesterol (TC), triglyceride (TG), HDL‐C and LDL‐C Mean (± SD, *n* = 6) and multiple comparison test results.

Groups	T. Cholesterol (mg/dL)	Triglyceride (mg/dL)	HDL (mg/dL)	LDL (mg/dL)
1 (HC group)	55.60 ± 4.45^d*^	47.60 ± 10.31^d*^	42.60 ± 6.69^b*^	17.00 ± 3.08^cd*^
2 (ACP group)	46.33 ± 11.53^d^	40.66 ± 5.68^d^	51.00 ± 4.56^b^	12.16 ± 1.47^c^
3 (TWR group)	318.16 ± 29.71^a^	1390.00 ± 181.93^a^	119.83 ± 53.89^a^	52.50 ± 32.72^a^
4 (*CL* group)	48.83 ± 2.48^d^	42.66 ± 6.68^d^	47.50 ± 4.96^b^	15.33 ± 1.96^cd^
5 (βS group)	50.16 ± 13.64^d^	44.00 ± 9.81^d^	48.83 ± 7.93^b^	15.33 ± 3.14^cd^
6 (CL + TWR group)	248.50 ± 21.13^b^	1160.33 ± 177.54^b^	113.50 ± 33.02^a^	40.66 ± 28.86^abc^
7 (βS + TWR group)	274.83 ± 6.73^b^	1211.33 ± 143.31^ab^	119.00 ± 26.55^a^	27.83 ± 17.85^abc^
8 (ACP + TWR group)	154.50 ± 18.87^c^	947.16 ± 63.27^c^	106.16 ± 27.20^a^	42.50 ± 37.95^ab^

Abbreviations: ACP, Anacetrapib; CL, *C. longiflorus*; HC, Healthy control; TWR, Triton WR‐1339; βS, β‐ sitosterol.

*While the difference between the group averages shown with the same letter is not significant (*p* > .05), the difference between the group averages shown with different letters is significant (*p* < .05).

#### 3.1.1 Total cholesterol (TC)

When the TC data was examined, the highest TC level among all application groups was seen in group 3, the group in which TWR was applied alone (318.16 ± 29.71). TC level seen in this group was statistically significantly higher than all treatment groups (*p* < .05). This result is an indication that TWR was used for positive control makes rats used in group 3 hyperlipidemic. The lowest TC level was measured in the rat group (group 2) in which ACP, which is another positive control and used as a cholesterol drug, was applied alone (46.33 ± 11.53). This result is proof that ACP shows its expected effect before the experiment is performed. However, when the decrease in TC level seen in group 2 was evaluated statistically, it was not much different from the TC values measured in group 1, group 4 and group 5 (*p* > .05). In other words, the healthy control, CL, βS and ACP application groups yielded similar results in terms of TC. Still, to evaluate, the ranking for these four groups in terms of TC was ACP group <CL group <βS group <HC group. In other words, the group in which the plant extract (CL) we used was applied alone reduced the TC level almost as much as in the drug group. When the data obtained from the application groups (group 6, group 7 and group 8) that we carried out to eliminate the high TC levels caused by TWR and constitute the second phase of our study, it was determined that the high TC levels caused by TWR were statistically significantly reduced in all three groups (*p* < .05). When these three groups were compared among themselves, it was observed that group 6 and group 7 showed similar results, and group 8, showing lower TC levels, was observed to be statistically separated from these two groups (*p* < .05). The order of the groups (groups 3, 6, 7 and 8) that constitute the synergistic effect part of the study in terms of TC was as follows (TWR group >βS group + TWR >CL + TWR group >ACP + TWR group). In other words, in rats whose TC level was increased by applying TWR, the decrease in TC level was higher in the group in which the plant extract was applied with TWR (TWR + CL) (248.50 ± 21.13) than the group in which βS was applied with TWR (TWR + βS). However, the decline was not as sharp as in group 8 (154.50 ± 18.87).

#### 3.1.2 Triglyceride (TG)

When another parameter, TG data, was examined, it was observed that the results were parallel with TC data, although there were some minor differences (Table 1 and Figure 1). When the TG data was examined, the highest TG level among all application groups was seen in group 3, the group in which TWR was applied alone (1390.00 ± 181.93). This result is another indicator that TWR used for positive control makes rats used in group 3 hyperlipidemic. The lowest TG level was measured in the rat group (group 2) where ACP, the reference drug group, was administered alone (39.64 ± 5.88). This result is proof that ACP shows its expected effect before the experiment is performed. HC, CL, βS and ACP groups showed similar results in terms of TG. The group in which the plant extract (CL) was applied alone reduced the TG level almost as much as in the drug group. When evaluated for both TC and TG, it was evaluated that the plant extract had a more effective anti‐lipidemic effect than βS used as an active ingredient. When the data obtained from the application groups (groups 6, group 7, and group 8) that we carried out to eliminate the high TG levels caused by TWR were examined, it was found that the high TG levels caused by TWR significantly decreased in the three groups (*p* < .05). When these three groups were compared among themselves, it was observed that group 6 and group 7 showed similar results, and group 8 showed lower TG levels and was statistically separated from these two groups (*p* < .05). The order of the groups (groups 3, 6, 7, and 8) that constitute the synergistic effect of the study in terms of TG was as follows (TWR group >βS + TWR group >CL + TWR group >ACP + TWR group). In other words, in rats whose TG level was increased by applying TWR, the decrease in TG level was higher in the TWR + CL group (1160.33 ± 177.54) compared to the TWR + βS group (1211.33 ± 143.31). However, the decrease was not as sharp as in group 8 (947.16 ± 63.27).

#### 3.1.3 HDL‐cholesterol (HDL‐C)

In group 1, the healthy control group, HDL‐C level was measured as 42.60 ± 6.69 mg/dL. All other groups were found above this value. The ranking of HDL‐C increase in the first stage of the study was group 4 <group 5 <group 2, while the ranking in the second stage was group 8 <group 6 <group 7 <group 3. After group 3, the value reached by HDL‐C in the βS + TWR group, which increased the amount of HDL‐C the most, was measured as 119.00 ± 26.55 mg/dL. There is a statistically significant increase especially in TWR, CL + TWR, βS + TWR, and ACP + TWR groups compared to the HC group (Table 1 and Figure 1).

#### 3.1.4 LDL‐cholesterol (LDL‐C)

There is a statistically insignificant (*p* > .05) decrease in LDL‐C levels obtained from the groups in which CL, βS, and ACP have applied alone when compared to the HC group, while a statistically significant increase in LDL‐C levels was observed in rats induced by TWR (52.50 ± 32.72) was detected (*p* < .05). In the study investigating the synergistic effect, statistically significant decreases were observed in all three groups (*p* < .05). Especially the βS + TWR group was the group that decreased LDL‐C levels the most statistically. Thus, this decrease was greater than the effect of the ACP + TWR group on LDL‐C. As a result, the order of lowering LDL‐C levels was determined as group 7 >group 6 >group 8 (Table 1 and Figure 1).

### 3.2 Serum parameters and histopathological changes

**FIGURE 2:**
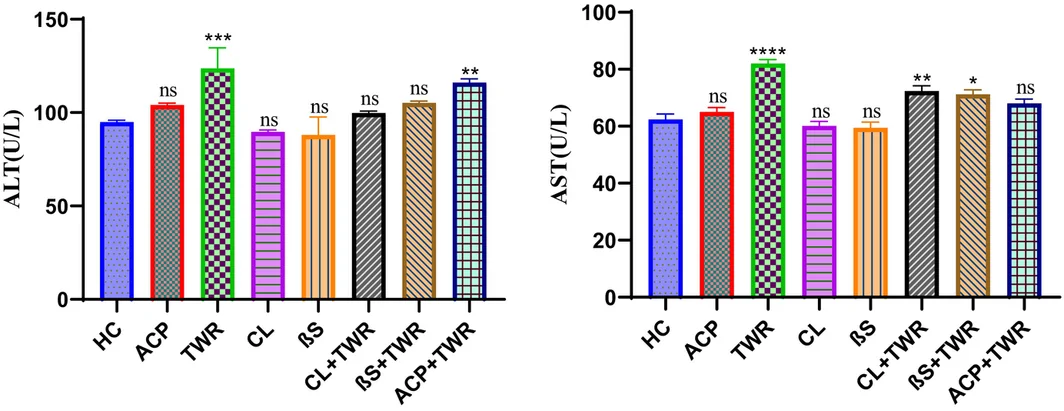
Effects of Triton Wr‐1339 (TWR), anacetrapib (ACP), *C. longiflorus* and β‐sitosterol on Alanine aminotransferase (ALT) and Aspartate aminotransferase (AST) in Wistar rats (Tukey's multiple range tests were used); *significant changes between control and treatment groups (ns: Not significant, **p < .01, ***p < .001, ****p < .0001).

The results obtained for all application groups of alanine aminotransferase (ALT) and aspartate aminotransferase (AST) are shown in Table 2 and Figure 2.

**TABLE 2:** Multiple comparison test results of ALT and AST levels and group mean data.

Groups	ALT(U/L)	AST(U/L)
1 (HC)	92.01 ± 8.93^cd*^	66.32 ± 8.52^b*^
2 (ACP)	102.02 ± 7.78^bcd^	69.22 ± 7.16^b^
3 (TWR)	121.30 ± 5.42^a^	85.54 ± 8.48^a^
4 (CL)	86.21 ± 7.91^d^	63.28 ± 6.54^b^
5 (βS)	87.51 ± 8.98^cd^	62.66 ± 5.95^b^
6 (CL + TWR)	98.05 ± 7.96^bcd^	75.22 ± 6.62^ab^
7 (βS + TWR)	102.28 ± 8.55^bc^	73.39 ± 6.15^ab^
8 (ACP + TWR)	113.55 ± 11.42^ab^	71.24 ± 6.13^b^

Abbreviations: ACP, Anacetrapib; CL, *C. longiflorus*; HC, Healthy control; TWR, Triton WR‐1339; βS, β‐ sitosterol.

*While the difference between the group averages shown with the same letter is not significant (*p* > .05), the difference between the group averages shown with different letters is significant (*p* < .05).

#### 3.2.1 Alanine aminotransferase (ALT)

When the ALT results obtained from the application groups were examined, it was seen that group 4 (CL) and group 5 (βS) decreased this value compared to the HC group (92.01 ± 8.93 U/L), and group 2 (ACP) increased this value. It was determined that ALT value increased significantly (121.30 ± 5.42 U/L) in group 3 (TWR) where only TWR was used (*p* < .05). It was determined that this increased value decreased in the synergistic effect groups group 6, group 7 and group 8. The CL + TWR group (98.05 ± 7.96 U/L) showed the most significant decrease.

#### 3.2.2 Aspartate aminotransferase (AST)

When the AST results obtained from the application groups were examined, the value of the healthy control group (66.32 ± 8.52 U/L), the values of AST decreases 63.28 ± 6.54 U/L, and 62.66 ± 5.95 U/L in the CL group and βS group, respectively. In other groups, it was above the HC group. The group that increased the AST value the most was group 3 where TWR was applied alone; there are no statistically significant decreases in group 6, group 7, and group 8, which were compared to group 3 (*p* > .05). Only this decrease in group 8 was statistically significant (*p* < .05).

#### 3.2.3 Histopathological changes

**FIGURE 3:**
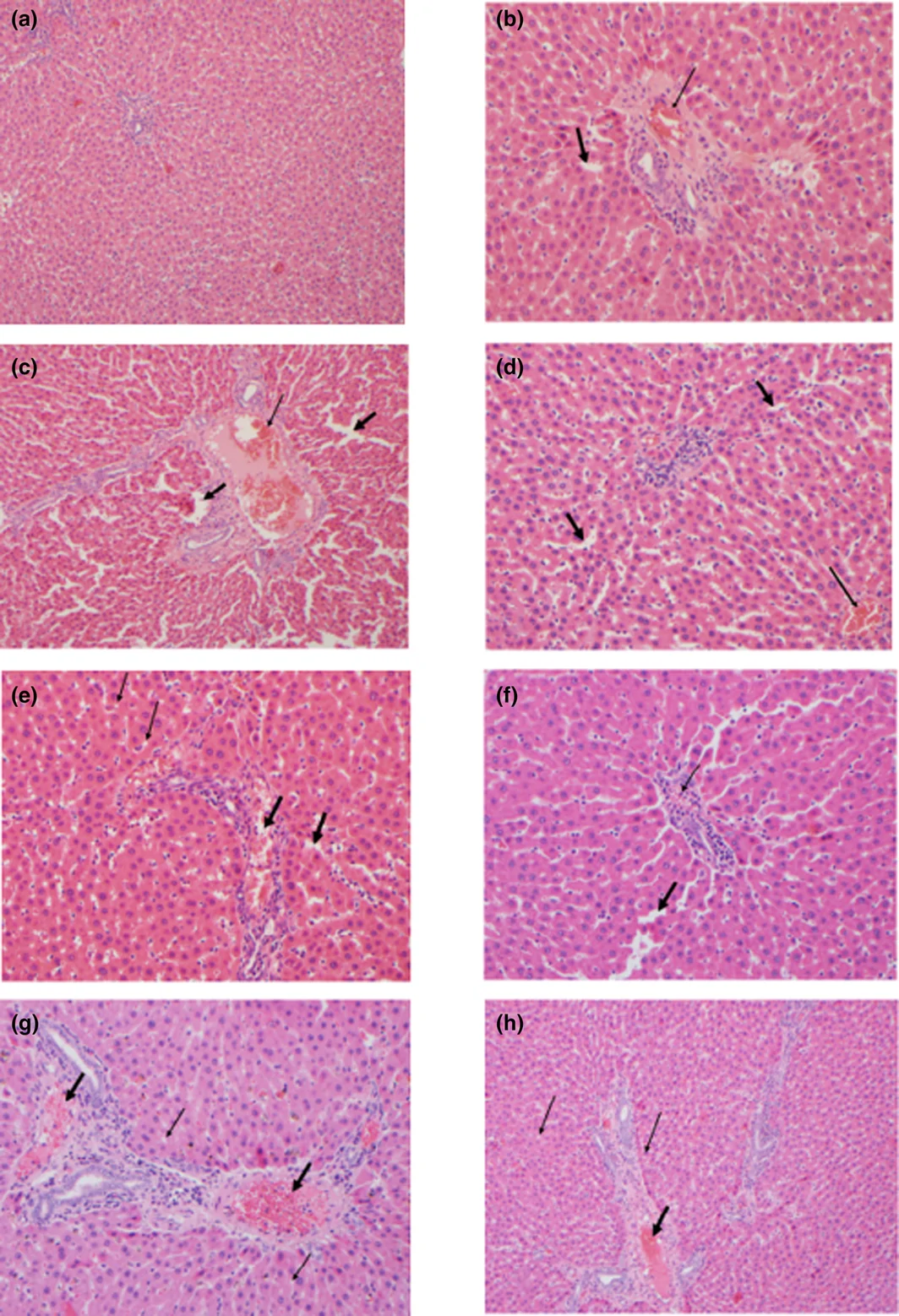
Effects of Triton Wr‐1339 (TWR), anacetrapib (ACP), *C. longiflorus* (CL) and β‐sitosterol (βS) on liver tissue in Wistar rats. (a) Liver, HC group; (b) ACP group: Mild liver damage (mild occlusion‐thin arrow and sinusoidal dilatation‐thick arrow). (c) TWR group: Severe liver injury (pronounced occlusion‐thin arrow and sinusoidal dilatation‐thick arrows) (d) CL group: Mild liver injury (mild occlusion‐thin arrow and sinusoidal dilatation‐thick arrows); (e) βS group: Moderate liver damage (a focal fat change with microdroplets‐thin arrows, moderate occlusion and sinusoidal dilatation‐thick arrows); (f) CL + TWR group: Mild liver damage (mild occlusion‐thin arrow and sinusoidal dilatation‐thick arrow). There is no fat change in hepatocytes; (g) βS + TWR group: Moderate liver damage (a focal fat exchange with microdroplets‐thin arrows and moderate occlusion thick arrows); (h) ACP + TWR group: Moderate liver damage (a focal fat exchange with microdroplets‐thin arrows and moderate occlusion thick arrows).

Figure 3 shows histopathological examination of liver tissues of Wistar rats by H&E staining in all treatment groups. When the data shown in Figure 3 were examined, it was determined that mild liver damage occurred in group 2 and group 4 male rats in which ACP and CL were administered alone. This slight damage in both groups; It was defined as some obstruction and sinusoidal dilatation. Moderate liver damage was detected in group 5, in which βS was administered alone. Damage in this group was defined as a focal oil change with microdroplets, moderate obstruction and sinusoidal dilatation. When CL, βS and ACP were applied together with TWR to male rats who became hyperlipidemic as a result of TWR application, the severe damage in group 3, in which TWR was applied alone, resulted in mild liver damage in the CL + TWR group (group 6); It was determined that the βS + TWR group (group 7) and ACP + TWR group (group 8) transformed into moderate liver damage. As a result, the combination of TWR and plant extract provided the most effective protection on the liver in male rats.

### 3.3 Cholesteryl ester transfer protein (CETP)

**FIGURE 4:**
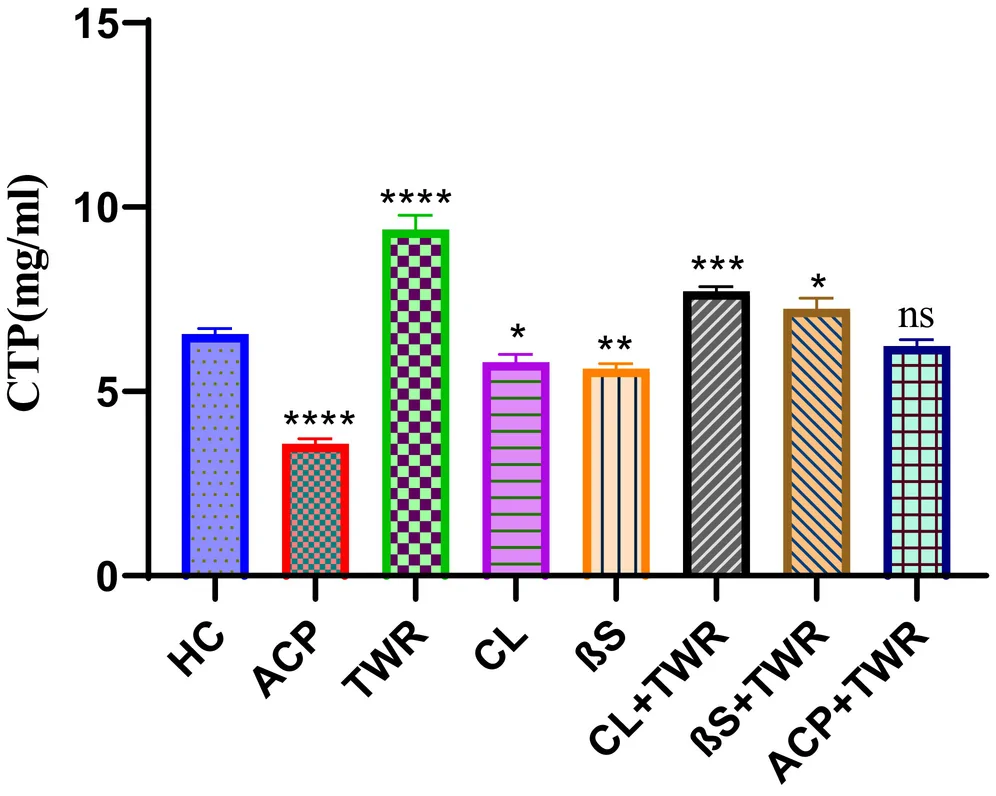
Effects of Triton Wr‐1339 (TWR), anacetrapib (ACP), *C. longiflorus* (CL) and β‐sitosterol (βS) on CETP levels in Wistar rats (Tukey's multiple range tests were used); *significant changes between control and treatment groups (ns: Not significant, **p < .01, ***p < .001, ****p < .0001).

When Table 3 and Figure 4 are examined, it can be seen that the CETP value was significantly inhibited in the groups in which CL, βS and ACP were administered alone (*p* < .05). While the CETP value was 6.36 ± 0.25 μg/mL in the HC group, it was measured as 3.57 ± 0.23 μg/mL in group 2. The CETP value reached the highest level in the TWR group, and it was 9.38 ± 0.31 μg/mL. In the groups induced by TWR, the ACP group achieved the most effective inhibition, reaching 6.42 ± 0.32 μg/mL, almost reaching the value of the HC group. Inhibition values of CL (group 6) and βS (group 7) were close to the ACP (group 8). (Table 3).

**TABLE 3:** Multiple comparison test results of CETP level values and group mean data.

Groups	CETP (μg/mL)
1 (HC)	6.36 ± 0.25c*
2 (ACP)	3.57 ± 0.23^e^
3 (TWR)	9.38 ± 0.31^a^
4 (*CL*)	5.75 ± 0.15^d^
5 (βS)	5.61 ± 0.20^d^
6 (CL + TWR)	7.64 ± 0.27^b^
7 (βS + TWR)	7.38 ± 0.38^b^
8 (ACP + TWR)	6.42 ± 0.32^c^

Abbreviations: ACP, Anacetrapib; CL, *C. longiflorus*; HC, Healthy control; TWR, Triton WR‐1339; βS, β‐ sitosterol.

*While the difference between the group averages shown with the same letter is not significant (*p* > .05), the difference between the group averages shown with different letters is significant (*p* < .05).

## 4 DISCUSSION

Triton WR‐1339 (Tyloxapol or an oxyethylated tertiary octyl phenol‐formaldehyde polymer) is a nonionic surfactant and has been proposed in many studies as suitable for inducing acute HL in animal models in order to screen natural or chemical drugs (Janicki & Aron, 1962). In many studies, HL application with TWR is similar to our study (Khlifi et al., 2019; Rony et al., 2014). This method provides the following advantages over the other method: It saves considerable time and cost. Additionally, similar results were obtained in comparison studies with other methods (Bouhlali et al., 2020). Its function is to cause a significant increase in hepatic cholesterol biosynthesis by stimulating the activity of the enzyme HMG‐CoA reductase. In addition, it inhibits the activity of the lipoprotein lipase enzyme and causes the accumulation of total cholesterol, triglycerides and very‐low‐density lipoprotein (VLDL‐C) in the plasma. In this study, a rat model with HL induced by TWR induction was used. This model is often applied for a variety of purposes (Hall et al., 2000) and it has been used specifically to screen for natural or chemical hypolipidemic drugs (Schurr et al., 1972). Twenty hours after TWR administration, the plasma total cholesterol and triglyceride levels reach their maximum levels and then decrease to normal values (Rony et al., 2014).

In the current study, the plasma lipid profile of rats in the healthy control group did not change during the experimental period. However, an intraperitoneal injection of triton resulted in high lipid concentrations in plasma. After 24 h of triton injection, a significant increase (*p* < .05) in TC, TG, LDL‐C and HDL‐C levels was noted compared to HC (Table 1 and Figure 1). The increase in serum TC and LDL‐C levels causes lipid atherogenesis, which may be the most important factor triggering the development of insulin resistance, atherogenic risk and metabolic syndrome (Khlifi et al., 2019). Injection of TWR into rats increases blood cholesterol and triglyceride levels and consequently increases the amount of VLDL‐C secreted by the liver. The antihyperlipidemic effect of CL extract (150 mg/kg) and βS (0.4%), in experimental models of HL, was investigated compared to a standard drug; anacetrapib (0.2 mg/kg). Administration of CL extract (150 mg/kg), βS (0.4%) and anacetrapib (0.2 mg/kg) by oral route in male rats resulted in a significant decrease in levels of serum TC, TG and LDL‐C compared to the hyperlipidemic group (TWR) (*p* < .05).

There is no reference in the literature related to the cholesterol‐lowering activity of CL extract. Cholesterol‐lowering functional foods either reduce cholesterol absorption and cholesterol synthesis, or increase cholesterol catabolism by converting cholesterol to bile acids for elimination via the bile duct (Chen et al., 2014). Our extract may have shown cholesterol‐lowering activity, possibly through the above‐menioned mechanisms. Also, the hypocholesterolemia effect of CL extract can be attributed to βS, which is often used as a cholesterol‐lowering functional plant sterol added to foods such as margarine and vegetable oil. It has been reported that phytosterols containing βS, when taken as supplements, can lower blood cholesterol concentration and reduce the risk of heart disease (Klingberg et al., 2008). In addition, many researchers have demonstrated the efficacy and safety of βS as a blood cholesterol‐lowering agent, not only for pharmaceuticals, but also as a food ingredient (Salehi et al., 2021). The present results clearly showed that CL plant extract is as effective as βS in lowering plasma cholesterol concentration, most likely mediated by reducing cholesterol absorption.

The liver, which is the main organ of the metabolic processes of the organism, is very affected by the complications that occur as a result of HL. The regression in liver functions and accompanying tissue damage, which can occur for many different reasons, can be determined by measuring the changes in enzyme parameters such as ALT, AST and LDH, which are produced and metabolized by the liver (Palabiyik et al., 2023). In addition, a significant increase in ALT and AST in the blood is a sign of serious damage to the liver tissue membranes (Chidambaram & Venkatraman, 2010). In previous studies, it has been reported that liver function enzymes such as ALT and AST increase after TWR application (Chen et al., 2014). In the current study, it was determined that intraperitoneal injection of TWR caused a significant increase in plasma ALT and AST levels compared to the HC group (Table 2 and Figure 2). However, there was no statistically significant change in ALT and AST levels in the groups (Askin et al., 2022; Bouhlali et al., 2020; Bowman et al., 2017) in which ACP, CL extract and βS were applied compared to the healthy control group. It was also determined that CL extract and βS applied to the hyperlipidemic rats increased ALT and AST levels caused by TWR closer to the healthy control group. It should be noted that the protective effect of CL extract and BS was better than that of the ACP‐treated group, especially for the ALT value. These data demonstrate the protective effect of CL and βS against liver injury during HL.

It has been reported in previous studies that HL causes changes in liver tissue morphology, and especially TWR increases the necrosis of cells around the central vessels in the liver, causing portal inflammation and sinusoidal congestion. (Garg et al., 2018). In the current study, effects such as significant congestion and sinusoidal dilatation, which indicate severe liver injury, were observed in the TWR group. In addition, in liver tissue; significant decrease in the histopathological changes observed in the TWR + CL and TWR + βS groups compared to the the TWR group demonstrated the histoprotective effectiveness of the use of CL and βS.

We talked about the role of CETP inhibitors in the management of high cholesterol in the Introduction. We have also mentioned that it is possible to use inhibition of CETP in determining the potential of some natural compounds to be target drugs for use in the treatment of cardiovascular diseases. We designed the present study to implement this strategy. In other words, we evaluated whether CL extract and βS would inhibit CETP with the reference drug, ACP. CL and βS caused inhibition of CETP both when administered alone and in combination with TWR. Let's also mention here that βS causes more effective inhibition. A study on the inhibition of CETP by both CL extract and βS was not found in the literature. However, there are studies with similar plants and compounds (Kwon et al., 2003; Liu & Yeh, 2002).

## 5 CONCLUSIONS

It was concluded that CL extract and βS have similar cholesterol‐lowering activity and this is mediated by decreased cholesterol absorption and synthesis and increased sterol excretion mechanisms. In particular, we can attribute the above‐mentioned cholesterol‐reducing properties of CL plant extract and βS to their contribution to CETP inhibition. Our results show that CL extract and βS can be used in diseases associated with HL due to their properties such as lowering cholesterol, protecting the liver against lipid damage, and having a similar effect to the reference drug (ACP).

## 6 THE LIMITATION OF THE PRESENT STUDY

This study has potential limitations. It is possible to evaluate these limitations in two categories: methodology and research process. Methodologically, the lack or scarcity of previous research studies on the subject can be said to be the most important limitation of the presented study. Limitations of the research process include access to information, longitudinal effects, cultural and other biases, language fluency, and time constraints.

## AUTHOR CONTRIBUTIONS

**Seda Askin:** Conceptualization (lead); investigation (lead); methodology (lead); project administration (lead); resources (lead); writing – original draft (equal); writing – review and editing (equal). **Fatma Zuhal Umudum:** Writing – original draft (equal); writing – review and editing (equal).

## CONFLICT OF INTEREST STATEMENT

The authors declare that they have no conflicts of interest with the contents of this article.

## Data Availability

Data available on request from the authors.

## References

[fsn34471-bib-0001] Askin, H. , Yilmaz, B. , Bakirci, S. , & Ayar, A. (2018). Simultaneous determination of α‐amyrin and β‐sitosterol in Centranthus longiflorus Stev. Subsp. longiflorus Stev and iris taochiaWoronow ex Grossh by GC‐MS method. Progress in Nutrition, 20(1‐S), 209–217.

[fsn34471-bib-0002] Askin, S. , Askin, H. , Dursun, E. , Palabiyik, E. , Uguz, H. , Cakmak, Ö. , & Koc, K. (2022). The hepato‐renal protective potential of walnut seed skin extract against acute renal ischemia/reperfusion damage. Cytokine, 153, 155861. 10.1016/j.cyto.2022.155861 35306426

[fsn34471-bib-0003] Attardo, S. , Musumeci, O. , Velardo, D. , & Toscano, A. (2022). Statins neuromuscular adverse effects. International Journal of Molecular Sciences, 23(15), 8364. 10.3390/ijms23158364 PMC9369175 35955495

[fsn34471-bib-0004] Bouhlali, E. , Hmidani, A. , Bourkhis, B. , Khouya, T. , Harnafi, H. , Zegzouti, Y. F. , & Alem, C. (2020). Effect of Phoenix dactylifera seeds (dates) extract in triton WR‐1339 and high fat diet induced hyperlipidaemia in rats: A comparison with simvastatin. Journal of Ethnopharmacology, 259, 112961. 10.1016/j.jep.2020.112961 32423881

[fsn34471-bib-0005] Bowman, L. , Hopewell, J. C. , Chen, F. , Wallendszus, K. , & Stevens, W. (2017). Effects of anacetrapib in patients with atherosclerotic vascular disease. Journal of Vascular Surgery, 67(1), 356. 10.1056/NEJMoa1706444 28847206

[fsn34471-bib-0006] Chen, J. , Jiao, R. , Jiang, Y. , Bi, Y. , & Chen, Z.‐Y. (2014). Algal sterols are as effective as β‐sitosterol in reducing plasma cholesterol concentration. Journal of Agricultural and Food Chemistry, 62(3), 675–681. 10.1021/jf404955n 24380496

[fsn34471-bib-0007] Chen, S. , Wang, R. , Cheng, M. , Wei, G. , Du, Y. , Fan, Y. , Li, J. , Li, H. , & Deng, Z. (2020). Serum cholesterol‐lowering activity of β‐sitosterol laurate is attributed to the reduction of both cholesterol absorption and bile acids reabsorption in hamsters. Journal of Agricultural and Food Chemistry, 68(37), 10003–10014. 10.1021/acs.jafc.0c04386 32811147

[fsn34471-bib-0008] Chidambaram, J. , & Venkatraman, A. C. (2010). Cissus quadrangularis stem alleviates insulin resistance, oxidative injury and fatty liver disease in rats fed high fat plus fructose diet. Food and Chemical Toxicology, 48(8–9), 2021–2029. 10.1016/j.fct.2010.04.044 20450951

[fsn34471-bib-0707] Cinar, I. , Sirin, B. , Aydin, P. , Toktay, E. , Cadirci, E. , Halici, I. , Halici, Z. (2019). Ameliorative effect of gossypin against acute lung injury in experimental sepsis model of rats. Life Sci, 221, 327–334. 10.1016/j.lfs.2019.02.039. 30797018

[fsn34471-bib-0009] Das, G. , Shin, H.‐S. , Tundis, R. , Gonçalves, S. , Tantengco, O. A. G. , Campos, M. G. , Acquaviva, R. , Malfa, G. A. , Romano, A. , & Robles, J. A. H. (2021). Plant species of sub‐family Valerianaceae—A review on its effect on the central nervous system. Plants, 10(5), 846. 10.3390/plants10050846 PMC8144999 33922184

[fsn34471-bib-0010] Douglas, G. , & Channon, K. M. (2014). The pathogenesis of atherosclerosis. Medicine, 42(9), 480–484.

[fsn34471-bib-0011] Feng, S. , Dai, Z. , Liu, A. B. , Huang, J. , Narsipur, N. , Guo, G. , Kong, B. , Reuhl, K. , Lu, W. , & Luo, Z. (2018). Intake of stigmasterol and β‐sitosterol alters lipid metabolism and alleviates NAFLD in mice fed a high‐fat western‐style diet. Biochimica et Biophysica Acta (BBA)‐molecular and cell biology of Lipids, 1863(10), 1274–1284. 10.1016/j.bbalip.2018.08.004 PMC6226309 30305244

[fsn34471-bib-0012] Galicia‐Garcia, U. , Jebari, S. , Larrea‐Sebal, A. , Uribe, K. B. , Siddiqi, H. , Ostolaza, H. , Benito‐Vicente, A. , & Martín, C. (2020). Statin treatment‐induced development of type 2 diabetes: From clinical evidence to mechanistic insights. International Journal of Molecular Sciences, 21(13), 4725. 10.3390/ijms21134725 PMC7369709 32630698

[fsn34471-bib-0013] Garg, G. , Patil, A. , Singh, J. , Kaushik, N. , Praksah, A. , Pal, A. , & Chakrabarti, A. (2018). Pharmacological evaluation of convolvulus pluricaulis as hypolipidaemic agent in triton WR‐1339‐induced hyperlipidaemia in rats. Journal of Pharmacy and Pharmacology, 70(11), 1572–1580. 10.1111/jphp.13004 30182365

[fsn34471-bib-0014] George, M. , Selvarajan, S. , Muthukumar, R. , & Elangovan, S. (2015). Looking into the crystal ball—Upcoming drugs for dyslipidemia. Journal of Cardiovascular Pharmacology and Therapeutics, 20(1), 11–20. 10.1177/1074248414545127 25079474

[fsn34471-bib-0015] Gillett, R. C., Jr. , & Norrell, A. (2011). Considerations for safe use of statins: Liver enzyme abnormalities and muscle toxicity. American Family Physician, 83(6), 711–716. 21404982

[fsn34471-bib-0016] Hall, J. A. , Gradin, J. L. , Andreasen, C. B. , & Wander, R. C. (2000). Use of a nonionic detergent (triton WR 1339) in healthy cats to assess hepatic secretion of triglyceride. American Journal of Veterinary Research, 61(8), 941–950. 10.2460/ajvr.2000.61.941 10951988

[fsn34471-bib-0017] Janicki, B. W. , & Aron, S. A. (1962). Effect of triton WR‐1339 on lipoproteins and lipoprotein lipase of Guinea pig plasma. Proceedings of the Society for Experimental Biology and Medicine, 109(3), 507–509. 10.3181/00379727-109-27250 14451114

[fsn34471-bib-0018] Khlifi, R. , Lahmar, A. , Dhaouefi, Z. , Kalboussi, Z. , Maatouk, M. , Kilani‐Jaziri, S. , Ghedira, K. , & Chekir‐Ghedira, L. (2019). Assessment of hypolipidemic, anti‐inflammatory and antioxidant properties of medicinal plant Erica multiflora in triton WR‐1339‐induced hyperlipidemia and liver function repair in rats: A comparison with fenofibrate. Regulatory Toxicology and Pharmacology, 107, 104404. 10.1016/j.yrtph.2019.104404 31199997

[fsn34471-bib-0019] Klingberg, S. , Ellegård, L. , Johansson, I. , Hallmans, G. , Weinehall, L. , Andersson, H. , & Winkvist, A. (2008). Inverse relation between dietary intake of naturally occurring plant sterols and serum cholesterol in northern Sweden. The American Journal of Clinical Nutrition, 87(4), 993–1001. 10.1093/ajcn/87.4.993 18400724

[fsn34471-bib-0020] Kotan, R. , Cakir, A. , Dadasoglu, F. , Aydin, T. , Cakmakci, R. , Ozer, H. , Kordali, S. , Mete, E. , & Dikbas, N. (2010). Antibacterial activities of essential oils and extracts of Turkish Achillea, Satureja and thymus species against plant pathogenic bacteria. Journal of the Science of Food and Agriculture, 90(1), 145–160. 10.1002/jsfa.3799 20355025

[fsn34471-bib-0021] Kuruüzüm‐Uz, A. , Güvenalp, Z. , Demirezer, L. Ö. , Bergère, I. , Ströch, K. , & Zeeck, A. (2002). 4′‐Deoxy iridoid glycosides from Centranthus longiflorus. Phytochemistry, 61(8), 937–941. 10.1016/s0031-9422(02)00476-4 12453521

[fsn34471-bib-0022] Kwon, M.‐J. , Song, Y.‐S. , Choi, M.‐S. , Park, S.‐J. , Jeong, K.‐S. , & Song, Y.‐O. (2003). Cholesteryl ester transfer protein activity and atherogenic parameters in rabbits supplemented with cholesterol and garlic powder. Life Sciences, 72(26), 2953–2964. 10.1016/s0024-3205(03)00234-0 12706483

[fsn34471-bib-0023] Liu, L. , & Yeh, Y.‐Y. (2002). S‐alk (en) yl cysteines of garlic inhibit cholesterol synthesis by deactivating HMG‐CoA reductase in cultured rat hepatocytes. The Journal of Nutrition, 132(6), 1129–1134. 10.1093/jn/132.6.1129 12042421

[fsn34471-bib-0024] Mansi, I. A. , Chansard, M. , Lingvay, I. , Zhang, S. , Halm, E. A. , & Alvarez, C. A. (2022). Statins and renal disease progression, ophthalmic manifestations, and neurological manifestations in veterans with diabetes: A retrospective cohort study. PLoS One, 17(7), e0269982. 10.1371/journal.pone.0269982 PMC9302779 35862466

[fsn34471-bib-0025] Masson, W. , Lobo, M. , Siniawski, D. , Huerín, M. , Molinero, G. , Valero, R. , & Nogueira, J. P. (2018). Therapy with cholesteryl ester transfer protein (CETP) inhibitors and diabetes risk. Diabetes & Metabolism, 44(6), 508–513. 10.1016/j.diabet.2018.02.005 29523487

[fsn34471-bib-0026] Mohammadpour, A. H. , & Akhlaghi, F. (2013). Future of cholesteryl ester transfer protein (CETP) inhibitors: A pharmacological perspective. Clinical Pharmacokinetics, 52, 615–626. 10.1007/s40262-013-0071-8 PMC3720705 23658137

[fsn34471-bib-0027] Nurmohamed, N. S. , Ditmarsch, M. , & Kastelein, J. J. P. (2022). Cholesteryl ester transfer protein inhibitors: From high‐density lipoprotein cholesterol to low‐density lipoprotein cholesterol lowering agents? Cardiovascular Research, 118(14), 2919–2931. 10.1093/cvr/cvab350 PMC9648826 34849601

[fsn34471-bib-0028] Palabiyik, E. , Sulumer, A. N. , Uguz, H. , Avci, B. , Askin, S. , & Askin, H. (2023). Walnut fruit diaphragm ethanol extract ameliorates damage due to triton WR‐1339‐induced hyperlipidemia in rats. European Journal of Lipid Science and Technology, 126, e2300105.

[fsn34471-bib-0029] Richardson, I. B. K. (1975). A revision of the genus Centranthus DC.(Valerianaceae). Botanical Journal of the Linnean Society, 71(3), 211–234.

[fsn34471-bib-0030] Rojas‐Fernandez, C. , Hudani, Z. , & Bittner, V. (2015). Statins and cognitive side effects: What cardiologists need to know. Cardiology Clinics, 33(2), 245–256. 10.1016/j.ccl.2015.02.008 25939297

[fsn34471-bib-0031] Rony, K. A. , Ajith, T. A. , Nima, N. , & Janardhanan, K. K. (2014). Hypolipidemic activity of Phellinus rimosus against triton WR‐1339 and high cholesterol diet induced hyperlipidemic rats. Environmental Toxicology and Pharmacology, 37(2), 482–492. 10.1016/j.etap.2014.01.004 24561532

[fsn34471-bib-0032] Salehi, B. , Quispe, C. , Sharifi‐Rad, J. , Cruz‐Martins, N. , Nigam, M. , Mishra, A. P. , Konovalov, D. A. , Orobinskaya, V. , Abu‐Reidah, I. M. , & Zam, W. (2021). Phytosterols: From preclinical evidence to potential clinical applications. Frontiers in Pharmacology, 11, 599959. 10.3389/fphar.2020.599959 PMC7841260 33519459

[fsn34471-bib-0033] Schmidt, A. F. , Hunt, N. B. , Gordillo‐Marañón, M. , Charoen, P. , Drenos, F. , Kivimaki, M. , Lawlor, D. A. , Giambartolomei, C. , Papacosta, O. , & Chaturvedi, N. (2021). Cholesteryl ester transfer protein (CETP) as a drug target for cardiovascular disease. Nature Communications, 12(1), 5640. 10.1038/s41467-021-25703-3 PMC8463530 34561430

[fsn34471-bib-0034] Schurr, P. E. , Schultz, J. R. , & Parkinson, T. M. (1972). Triton‐induced hyperlipidemia in rats as an animal model for screening hypolipidemic drugs. Lipids, 7(1), 68–74. 10.1007/BF02531272 5013174

[fsn34471-bib-0035] Simic, I. , & Reiner, Z. (2015). Adverse effects of statins‐myths and reality. Current Pharmaceutical Design, 21(9), 1220–1226. 10.2174/1381612820666141013134447 25312733

[fsn34471-bib-0036] Suleyman, H. , Guvenalp, Z. , Kizilkaya, M. , & Demirezer, L. O. (2007). Sedative effect of Centranthus longiflorus ssp. Longiflorus in rats and the influence of adrenalectomy on its effect. Yakugaku Zasshi, 127(8), 1263–1265. 10.1248/yakushi.127.1263 17666879

[fsn34471-bib-0037] Tan, E. Y. , Hartmann, G. , Chen, Q. , Pereira, A. , Bradley, S. , Doss, G. , Zhang, A. S. , Ho, J. Z. , Braun, M. P. , & Dean, D. C. (2010). Pharmacokinetics, metabolism, and excretion of anacetrapib, a novel inhibitor of the cholesteryl ester transfer protein, in rats and rhesus monkeys. Drug Metabolism and Disposition, 38(3), 459–473. 10.1124/dmd.109.028696 20016052

[fsn34471-bib-0038] Tsymbalyuk, Z. M. , Ivanova, D. , & Nitsenko, L. M. (2021). Taxonomic significance of palynomorphological characteristics of selected Centranthus (Caprifoliaceae) species. Hacquetia, 20(2), 243–256.

[fsn34471-bib-0040] Wang, H. , Wang, Z. , Zhang, Z. , Liu, J. , & Hong, L. (2023). Beta‐sitosterol as a promising anticancer agent for chemoprevention and chemotherapy: Mechanisms of action and future prospects. Advances in Nutrition, 14, 1085–1110. 10.1016/j.advnut.2023.05.013 PMC10509430 37247842

[fsn34471-bib-0041] Wang, S. , Ye, K. , Shu, T. , Tang, X. , Wang, X. J. , & Liu, S. (2019). Enhancement of Galloylation efficacy of Stigmasterol and β‐Sitosterol followed by evaluation of cholesterol‐reducing activity. Journal of Agricultural and Food Chemistry, 67(11), 3179–3187. 10.1021/acs.jafc.8b06983 30827096

[fsn34471-bib-0042] Wang, S. , Zhao, W. , Sun, L. , Xiao, S.‐M. , Lin, S. , Zhao, J. , Xiao, H. , Xing, X. , Lao, X. Q. , & Chen, Y.‐M. (2021). Independent and opposing associations of dietary phytosterols intake and PLCE1 rs2274223 polymorphisms on esophageal squamous cell carcinoma risk. European Journal of Nutrition, 60(8), 4357–4366. 10.1007/s00394-021-02561-9 34046701

[fsn34471-bib-0043] Zengin, G. , Nithiyanantham, S. , Locatelli, M. , Ceylan, R. , Uysal, S. , Aktumsek, A. , Selvi, P. K. , & Maskovic, P. (2016). Screening of in vitro antioxidant and enzyme inhibitory activities of different extracts from two uninvestigated wild plants: Centranthus longiflorus subsp. longiflorus and Cerinthe minor subsp. auriculata. European Journal of Integrative Medicine, 8(3), 286–292.

